# Applying dimensional psychopathology: transdiagnostic associations among regional homogeneity, leptin and depressive symptoms

**DOI:** 10.1038/s41398-020-00932-0

**Published:** 2020-07-22

**Authors:** Yan-ge Wei, Jia Duan, Fay Y. Womer, Yue Zhu, Zhiyang Yin, Lingling Cui, Chao Li, Zhuang Liu, Shengnan Wei, Xiaowei Jiang, Yanbo Zhang, Xizhe Zhang, Yanqing Tang, Fei Wang

**Affiliations:** 1 Brain Function Research Section, the First Affiliated Hospital of China Medical University, 110001 Shenyang, Liaoning P.R. China; 2 Henan Key Laboratory of Immunology and Targeted Drugs, School of Laboratory Medicine, Xinxiang Medical University, 453003 Xinxiang, Henan P.R. China; 3 Department of Psychiatry, the First Affiliated Hospital of China Medical University, 110001 Shenyang, Liaoning P.R. China; 4 Department of Psychiatry, Washington University School of Medicine, St. Louis, MO 63130 USA; 5 Department of Radiology, the First Affiliated Hospital of China Medical University, 110001 Shenyang, Liaoning P.R. China; 6 School of Public Health, China Medical University, 110001 Shenyang, Liaoning P.R. China; 7 Department of Psychiatry, College of Medicine University of Saskatchewan, Ellis Hall, Royal University Hospital, Saskatoon, SK S7N 0W8 Canada; 8 Affiliated Nanjing Brain Hospital, Nanjing Medical University, 210029 Nanjing, Jiangsu P.R. China; 9 School of Biomedical Engineering and Informatics, Nanjing Medical University, 211166 Nanjing, Jiangsu P.R. China; 10 Department of Geriatrics, the First Affiliated Hospital of China Medical University, 110001 Shenyang, Liaoning P.R. China

**Keywords:** Neuroscience, Predictive markers

## Abstract

Dimensional psychopathology and its neurobiological underpinnings could provide important insights into major psychiatric disorders, including major depressive disorder, bipolar disorder and schizophrenia. In a dimensional transdiagnostic approach, we examined depressive symptoms and their relationships with regional homogeneity and leptin across major psychiatric disorders. A total of 728 participants (including 403 patients with major psychiatric disorders and 325 age–gender-matched healthy controls) underwent resting-state functional magnetic resonance imaging at a single site. We obtained plasma leptin levels and depressive symptom measures (Hamilton Depression Rating Scale (HAMD)) within 24 h of scanning and compared the regional homogeneity (ReHo), plasma leptin levels and HAMD total score and factor scores between patients and healthy controls. To reveal the potential relationships, we performed correlational and mediational analyses. Patients with major psychiatric disorders had significant lower ReHo in primary sensory and visual association cortices and higher ReHo in the frontal cortex and angular gyrus; plasma leptin levels were also elevated. Furthermore, ReHo alterations, leptin and HAMD factor scores had significant correlations. We also found that leptin mediated the transdiagnostic relationships among ReHo alterations in primary somatosensory and visual association cortices, core depressive symptoms and body mass index. The transdiagnostic associations we demonstrated support the common neuroanatomical substrates and neurobiological mechanisms. Moreover, leptin could be an important association among ReHo, core depressive symptoms and body mass index, suggesting a potential therapeutic target for dimensional depressive symptoms across major psychiatric disorders.

## Introduction

For over a century, the three major psychiatric disorders (MPDs), namely, major depressive disorder (MDD), bipolar disorder (BPD) and schizophrenia (SCZ), have primarily been considered as distinct disorders with separate mechanisms of disease. Researchers have studied MPDs as separate diagnostic entities. However, genetic susceptibility, metabolic disturbances, neural alterations and symptomatology across MPDs overlap, suggesting a transdiagnostic continuum of major endogenous psychoses^1–3^. The Research Domain Criteria has worked towards a dimensional transdiagnostic approach by integrating research from genomics, molecules, neural circuitry, psychological and clinical manifestations^4,5^. Most transdiagnostic studies have focussed on the differences; however, their neurobiological substrate remains poorly understood. Recent studies have documented that the dimensionality of clinical presentations may share common neurobiological associations^2,3,6–8^. Herein this dimensional transdiagnostic approach to explore the multilevel link might provide new insights into common neurobiological mechanisms and would therefore be useful for precision medicine across MPDs.

Depressive symptoms are common in all three MPDs. For instance, individuals at a high risk for SCZ with depressive symptoms are at an increased risk for progression to psychosis^9^. In a meta-analysis of 56 studies, depressive symptoms were associated with a high BPD risk. Approximately 40% of BPD cases were initially diagnosed as MDD, and 22% of MDD cases were are later diagnosed as BPD at up to 12–18 years of follow-up^10^. Collectively, depressive symptoms typically precede the onset of a more severe psychopathology in SCZ and BPD. On a dimensional scale, depressive symptoms may present disruptions within a specific neural circuitry across MPDs. However, its underlying neurobiological mechanism remains unclear.

One powerful tool to explore neural circuitry alterations is intrinsic resting-state functional connectivity, such as regional functional connectivity^11^. Regional homogeneity (ReHo) is a highly sensitive, reproducible and reliable index of regional functional connectivity^12^. ReHo is believed to reflect anatomical, morphological, and intrinsically geometric similarity in a local brain structure as well as a topology-functionality interplay. Accumulating evidence suggests that ReHo changes are associated with the pathophysiology of MPDs^11–16^. ReHo alterations in the prefrontal cortex, thalamus, right supplemental motor area and primary visual, auditory and motor cortices have been detected across SCZ, BPD and MDD. Moreover, a positive correlation has been found between ReHo in the left superior temporal gyrus and depressive symptoms in BPD^17^. A recent large, resting-state functional magnetic resonance imaging (fMRI) data set (709 patients with MDD and 725 healthy controls, including our data set) found that lower ReHo in the postcentral gyrus was associated with depressive symptoms in MDD^18^. Collectively, ReHo may be a transdiagnostic neurobiological substrate for evaluating the reproducible alterations underlying depressive symptoms.

In studying the dimensional underpinning of MPDs, we need to understand the molecular influences on neural circuitry. For decades, abnormal leptin levels have been detected in SCZ, BPD and MDD^19–21^. Leptin is an adipocyte-secreted hormone, primarily known for its role in energy regulation in appetite and body weight^22^. It enters the brain through a saturable, passive transport at the blood–brain barrier^23^; the human brain is a source of leptin in the plasma^24^. Leptin is associated with depressive symptoms and behaviours through various brain systems^25^. Animal studies demonstrated that the knockout of leptin receptors induced depression-related behaviours^26^. Leptin can affect the release, synthesis and metabolism of emotional mediators, including norepinephrine, 5-hydroxytryptamine and dopamine^22^; thus, it may be a possible transdiagnostic link across MPDs.

In this study, we developed a transdiagnostic approach to explore the neurobiological basis of dimensional psychopathology across MPDs. Using correlational and mediational analyses, we examined the relationship among molecular brain-depressive symptoms in MPDs. We hypothesised that plasma leptin levels and ReHo significantly correlate with depressive symptoms and leptin further mediates the association between ReHo and depressive symptoms.

## Materials and methods

### Participants

This study enrolled 728 individuals aged 13–55 years (127 with SCZ, 123 with BPD, 153 with MDD and 325 controls). We recruited the participants from the outpatient and inpatient units at the Department of Psychiatry of the First Affiliated Hospital of China Medical University and Shenyang Mental Health Centre in Shenyang, China. For those aged ≥18 years, 2 trained clinical psychiatrists independently confirmed their diagnoses by using the Structured Clinical Interview for Diagnostic and Statistical Manual of Mental Disorders, Fourth Edition (DSM-IV) Axis I Disorders (SCID). For patients aged <18 years, they used the Schedule for Affective Disorders and Schizophrenia for School-Age Children-Present and Lifetime Version. All the participants met the DSM-IV diagnostic criteria for SCZ, BPD or MDD without any other axis I disorder. We recruited the controls from the local area via advertisements and confirmed that they had no current or lifetime axis I disorder by using the SCID Non-Patient Version as well as no history of axis I disorders in their first-degree relatives. Exclusion criteria for all participants were as follows: (1) disordered eating and substance or alcohol abuse/dependence, (2) concomitant major medical disorders, (3) significant pathological changes identified on high-resolution T1- and T2-weighted MRI, (4) head trauma with loss of consciousness for ≥5 min and neurological disorders, and (5) MRI contraindications.

This study was approved by the Institutional Review Board of the China Medical University and was conducted in accordance with the Declaration of Helsinki. All participants provided written informed consent. All experiments and methods were carried out in accordance with approved regulations and guidelines.

### Clinical and cognitive assessment

We measured the weight and height of the subjects according to the written, standardised instructions provided in a manual. The following anthropometric measure was calculated according to standardised approaches: body mass index [BMI; calculated as weight divided by the square of height (kg/m^2^)]. Moreover, all participants completed the Hamilton Depression Rating Scale (HAMD), the Young Mania Rating Scale (YMRS) and Brief Psychiatric Rating Scale (BPRS).

### MRI acquisition

MRI scans were obtained using a 3.0-T GE Sigma system (Sigma EXCITE HDx; GE Healthcare, Milwaukee, MI, USA) with a standard eight-channel head coil at the First Affiliated Hospital of the China Medical University, Shenyang, China. Head motion was minimised with restraining foam pads provided by the manufacturer. All participants were instructed to be relaxed and keep their eyes closed without moving and falling asleep during the scan. The resting-state functional sequence was as follows: repetition time = 2000 ms, echo time = 30 ms, flip angle = 90°, field of view = 240 × 240 mm^2^, matrix = 64 × 64, 35 slices, slice thickness/gap = 3 mm/0 mm. The scan lasted for 6 min and 40 s.

### Data processing

The images were processed and analysed using the Statistical Parametric Mapping 8 (SPM8; http://www.fil.ion.ucl.ac.uk/spm) and Data Processing Assistant for R-functional MRI-fMRI (DPARSF; http://www.restfmri.net/forum/DPARSF) toolkits^27^. The first ten time points of functional images were discarded to ensure magnetisation stabilisation. Next, the remaining images were corrected for slice timing. The six-parameter rigid body transformation (three rotations and three translations) were used for image realignment and head motion correction. All subjects with a head motion >3.0° rotation and 3.0 mm translation were excluded. We normalised motion-corrected functional images to standard EPI template in Montreal Neurological Institute space and then resampled them to 3 × 3 × 3 mm^3^. At this stage, we removed the linear detrending to reduce the influence of increased MRI equipment temperature and demonstrated temporal band-pass filtering (0.01–0.08 Hz) to minimise high-frequency noise and the effect of low frequency. Then the nuisance signals including 24 head motion parameters, global mean, white matter and cerebrospinal fluid were regressed out from the data^18,28–31^. In addition, we utilised the mean framewise displacement (FD) to address the residual effects of motion on between-group differences. No significant differences for the mean FD were observed between patients with MPDs and controls (*t* = −0.867, *P* = 0.386). Then mean FD was set as a covariate in the statistical analyses to minimise head motion confounds^32,33^.

### Calculation of ReHo

To characterise the ReHo, we used an ReHo approach, which applies Kendall’s coefficient of concordance (KCC) to measure the degree of ReHo in resting-state fMRI. We developed ReHo maps for individual participants by calculating the KCC of the time series for a given voxel with regard to 26 neighbouring voxels^34^.The preprocessed individual 4D images are not spatially smoothed, considering that ReHo is an intrinsic smoothing computation across the neighbours in space. However, to improve the performance of group-level statistical comparisons, we smoothed all individual ReHo maps by using a 6-mm full-width half-maximum Gaussian filter.

### Measurement of plasma leptin levels

Five milliliters of venous blood samples were collected from 194 participants, centrifuged at 2000 rpm for 10 min and then stored at −80 °C. Plasma leptin levels were measured by the Human Premixed Multi-Analyte Kit (R&D Systems, Inc., Minneapolis, MN, USA) with the Human Magnetic Luminex Assay (Leptin [BR51]). The assay was performed in duplicate according to the manufacturer’s directions, and intra- and inter-assay coefficients of variation were <10% for leptin. Samples were randomised and the two operators were blinded to all clinical information. The assays were calibrated using standards; raw intensity measurements were converted to absolute concentrations by comparison with the standards. Those that fell below the minimum level of detection were assigned a value of minimum level of detection. Detailed information of this method can be found in Supplementary Materials.

### Statistical analysis

#### Statistical analyses of demographic and clinical characteristics

SCZ, BPD and MDD constituted the MPD group, and we conducted a series of analyses to compare MPDs and controls. The normality of continuous variables was tested using one-sample Kolmogorov–Smirnov (K-S) test. Group differences between continuous variables were tested using *t* test (normally distributed data) or Mann–Whitney *U* test (non-normally distributed data). Chi-square tests were used to determine differences between categorical variables. Plasma leptin levels were natural log-transformed to obtain normal distributions. Demographic and clinical data was analysed using the IBM SPSS Statistics for Windows, version 22.0 (IBM Corp., Armonk, NY, USA). The statistical significance was set as *P* < 0.05. To confirm the reliability of our data, effects of three potential confounders (i.e., duration, the first episode and medication) were examined (see Supplementary Data for full details).

#### HAMD factor analyses

To identify a parsimonious list of factors for the HAMD, we employed the exploratory factor analysis (EFA) and confirmatory factor analysis (CFA). We randomly divided all patients into two subsamples, namely, initial sample and replication sample, for EFA and CFA, respectively. We then employed Kaiser–Meyer–Olkin (KMO) measure of sampling adequacy and Bartlett’s test of sphericity to assess the appropriateness of factor analysis on the data. To estimate the internal consistency reliability, we calculated Cronbach’s alpha, which is the most widely used method, for all main factors and for each dimension. We selected the varimax rotation method to simplify the interoperability of the factor solution in the dimensional process. Furthermore, we used the identified EFA factors in subsequent analyses, as detailed subsequently. CFA for the HAMD can be found in Supplementary Materials.

#### Voxel-wise analyses of ReHo values

We used the DPABI to perform voxel-based two-sample *t* tests to compare ReHo values between the MPDs and controls, with the diagnostic group as an independent factor and age, gender and mean FD as covariates. A voxel-wised threshold was set at *P* < 0.001 with false discovery rate (FDR) correction in DPABI 4.1.

#### Correlation analyses

We conducted partial correlation analyses (two-tailed) to examine relationships with ReHo values in regions with significant between-group differences, plasma leptin levels, HAMD total scores, HAMD factor scores and BMI in the MPD groups. Age, gender, mean FD and medication were considered as covariates. In addition, to assess the effects of BMI on ReHo values, which were extracted from the regions showing significant differences, and plasma leptin levels in the controls, we performed partial correlation analyses (two-tailed) controlling for age, gender and mean FD. The significance level was set as *P* < 0.05, with FDR for multiple comparison correction.

#### Confounding effects

To assess reliability, we examined the influence of potential confounding variables (i.e., illness duration, the first episode and medication status). The details can be found in Supplementary Materials (see Supplementary Methods).

#### Mediation analyses

Once we identified significant ReHo-leptin-depressive symptom measures associated, we conducted mediation analyses to test whether leptin mediates the association between ReHo and depressive symptoms. On the basis of a standard three-variable path model, we performed mediation analyses by using the PROCESS for SPSS 22.0 statistical software with a 5000 bias-corrected bootstrap sample for significance testing. As mentioned, we treated age, gender, mean FD and medication as covariates. Statistical significance was achieved when 95% confidence intervals (CIs) did not include zero for the estimates of indirect effect^35–37^.

## Results

### Demographic and clinical characteristics

We found no significant differences in age, gender, handedness, weight, BMI and smoking between the MPDs and controls (*P* > 0.05). K-S test showed that plasma leptin levels in each group were normally distributed (*P* > 0.05), whereas age, BMI, illness duration, HAMD, YMRS and BPRS in each group failed to pass the normality test (*P* < 0.05). Table 1 presents detailed demographic and clinical data of the MPDs and controls, whereas Table S1 lists detailed characteristics of the SCZ, BPD and MDD groups.

**Table 1:** Participant demographic and clinical characteristics.

Characteristic	Group; mean ± SD or no. (%)		Statistics	*P* value
	Control (*n* = 325)	MPDs (*n* = 403)		
Demographic characteristics				
Age, years	29.24 (9.94)	28.47 (8.88)	1.091	0.27
Gender (male/female)	132/193	143/260	2.015	0.156
Right handedness	303 (93%)	360 (89%)	0.796	0.672
Weight^a^	62.41 (14.39)	62.90 (12.93)	−0.479	0.632
BMI (kg/m^2^)^a^	23.76 (26.60)	22.58 (3.90)	0.857	0.392
Smoking	37/188 (20%)	49/206 (24%)	0.971	0.324
Clinical characteristics				
First episode, yes	–	262 (65%)	–	–
Medication, yes	–	259 (64%)	–	–
Antidepressant	–	125 (31%)		
Mood stabiliser	–	70 (17%)	–	–
Antipsychotic	–	124 (31%)	–	–
Duration, months	–	32.98 (49.21)	–	–
HAMD^b^	1.27 (2.16)	13.79 (10.57)	−20.262	<0.001
YMRS^c^	0.25 (0.93)	2.97 (6.40)	−7.255	<0.001
BPRS^d^	18.42 (1.94)	28.90 (10.08)	−15.779	<0.001
Leptin (pg/ml)^e^	5463.13 (4055.93)	9510.14 (9539.14)	−4.011	<0.001
Leptin (log)^e^	3.57 (0.44)	3.78 (0.46)	−3.104	0.002

*BMI* body mass index, *BPRS* Brief Psychiatric Rating Scale, *HAMD* Hamilton Depression Scale, *YMRS* Young Mania Rating Scale.

^a^Control, *n* = 314; patients with major psychiatric disorders, *n* = 385.

^b^Control, *n* = 303; patients with major psychiatric disorders, *n* = 374.

^c^Control, *n* = 298; patients with major psychiatric disorders, *n* = 337.

^d^Control, *n* = 237; patients with major psychiatric disorders, *n* = 303.

^e^Control, *n* = 83; patients with major psychiatric disorders, *n* = 111.

### HAMD factor analyses

We analysed the HAMD factor to determine the best number of factors that described the scale. Bartlett’s test of sphericity was statistically significant (*P* < 0.001), and the KMO value was 0.946. The Cronbach’s alpha was 0.927, demonstrating a strong internal consistency. The data can be deemed to be suitable for EFA. Using the maximum variance method, we identified HAMD-17 items with 4 dimensions, accounting for 64.425% of the total variance in the initial sample. We also determined four-factor rotation to provide the optimal description of the HAMD scales. Then we labelled these 4 factors as psychological depressive symptoms (Factor 1), somatic depressive symptoms (Factor 2), insomnia (Factor 3) and mixed symptoms (Factor 4). EFA results can be found in Table S2. CFA results for the HAMD can be found in Supplementary Materials.

### ReHo values and plasma leptin levels across the diagnostic groups

**Fig. 1:**
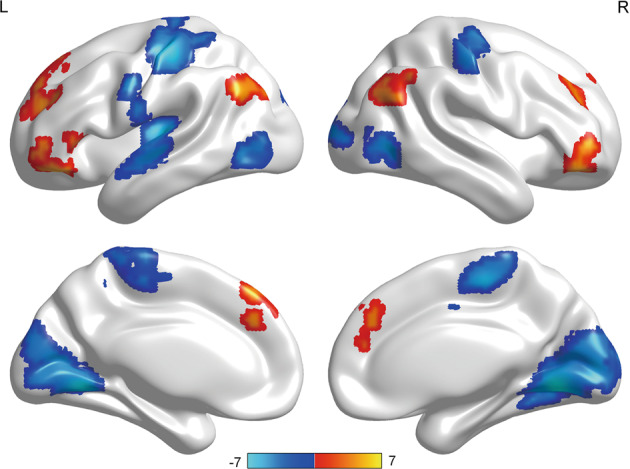
Significant differences in regional homogeneity between patients with MPDs and healthy controls. The significance was set at *P*_FDR_ < 0.05 with voxel *P* < 0.001. Red in the colour bar indicates relatively higher ReHo values; blue colour denotes relatively lower ReHo values in patients with MPDs. FDR false discovery rate, MPD major psychiatric disorders, ReHo regional homogeneity.

The MPD group exhibited lower ReHo in the bilateral primary somatosensory cortices, left primary auditory cortex, right primary visual cortex and bilateral visual association cortices. Primarily, ReHo was significantly higher in the bilateral orbitofrontal cortices, bilateral dorsolateral prefrontal cortices and bilateral angular gyri (Fig. 1 and Table 2). Specific ReHo values of the SCZ, BPD and MDD groups are listed in Supplementary Materials and Fig. S1. Compared with those in the controls, the leptin levels in patients with MPDs were significantly elevated (Table 1). Specific leptin levels of the SCZ, BPD and MDD groups can be found in Supplementary Materials, Fig. S2 and Table S1.

**Table 2:** Regional homogeneity values in brain regions showing significant group differences.

Brain regions	BA	Cluster size	Peak MNI coordinates			*F*-value
			*X*	*Y*	*Z*	
Patients’ group < control						
Right primary somatosensory cortex	1/2/3	211	51	−21	57	−4.6993
Left primary somatosensory cortex	1/2/3	746	−48	−27	54	−6.5973
Left primary auditory cortex	41	398	−39	−18	9	−6.2337
Right primary visual cortex	17/18/19	1300	15	−66	−3	−7.5493
Right visual association cortex	19/37	103	48	−69	0	−5.1931
Left visual association cortex	19/37	95	−48	−72	−6	−5.0662
Patients’ group > control						
Right orbital frontal cortex	11/47	150	42	39	−9	5.6068
Left orbital frontal cortex	11/47	182	−45	39	−15	4.9879
Right dorsolateral prefrontal cortex	9	83	33	45	30	4.087
Left dorsolateral prefrontal cortex	9	277	−21	48	21	5.2131
Right angular gyrus	39	112	42	−66	33	4.9476
Left angular gyrus	39	114	−51	−66	33	4.6568

Significant at *P* < 0.05 corrected and a corrected *P* < 0.001 at the voxel level using false discovery rate corrections for multiple comparisons.

*BA* Brodmann areas, *MNI* Montreal Neurological Institute.

### Correlations between ReHo values, plasma leptin levels and clinical variables

**Fig. 2:**
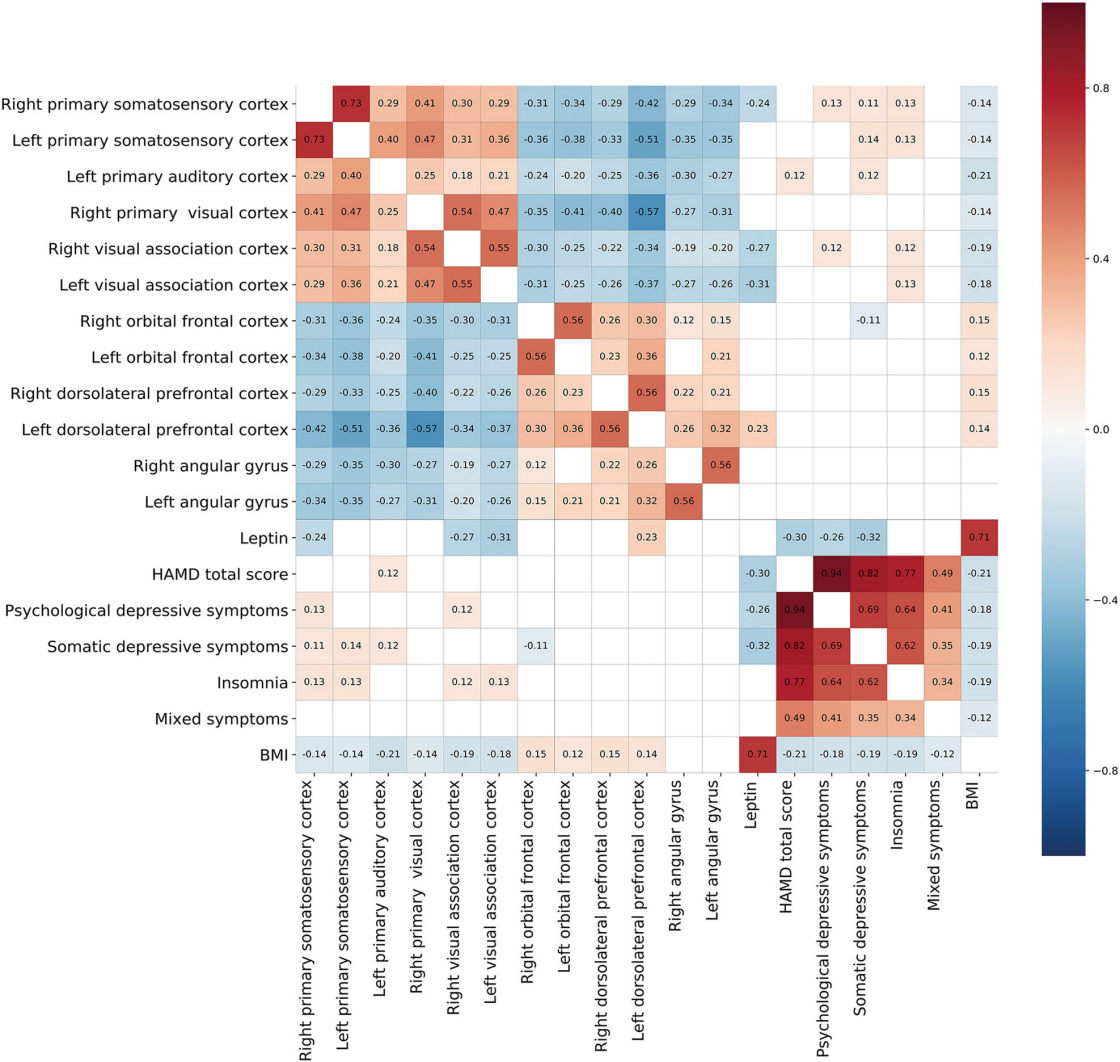
Correlation matrices. Correlation among ReHo within abnormal regions, plasma leptin levels, HAMD total score, HAMD factor scores, and BMI in patients with MPDs. Covariates were age, sex, mean FD, and medication. Statistical significance was set at *P*_FDR_ < 0.05 for multiple comparisons. The colour bar indicates correlation coefficient values (*R*); red and blue denote positive and negative relationships among MPDs, respectively. BMI body mass index, FDR false discovery rate, HAMD Hamilton Depression Scale, MPD major psychiatric disorder, ReHo regional homogeneity.

In the controls, ReHo values, plasma leptin levels and BMI did not significantly correlate (Table S3). ReHo correlated with plasma leptin levels, HAMD total score, HAMD factor scores and BMI after the FDR correction for multiple comparisons, as shown in partial correlation matrices (Fig. 2) and Tables S4–S7.

The correlated ReHo–leptin–HAMD score triplets were as follows: (ReHo in right primary somatosensory cortex–leptin: *r* = −0.245, *P* = 0.011; leptin–psychological depressive symptoms: *r* = −0.261, *P* = 0.007 and ReHo in right primary somatosensory cortex–psychological depressive symptoms: *r* = 0.129, *P* = 0.010); (ReHo in right visual association cortex–leptin: *r* = −0.270, *P* = 0.005; leptin–psychological depressive symptoms: *r* = −0.261, *P* = 0.007 and ReHo in right visual association cortex–psychological depressive symptoms: *r* = 0.115, *P* = 0.022); and (ReHo in right primary somatosensory cortex–leptin: *r* = −0.245, *P* = 0.011; leptin–somatic depressive symptoms: *r* = −0.317, *P* = 0.001 and ReHo in right primary somatosensory cortex–somatic depressive symptoms: *r* = 0.114, *P* = 0.023; Fig. 2; Tables S4–S6).

The correlated ReHo–leptin–BMI triplets were as follows: (ReHo in right primary somatosensory cortex–leptin: *r* = −0.245, *P* = 0.011; leptin–BMI: *r* = 0.710, *P* < 0.001 and ReHo in right primary somatosensory cortex–BMI: *r* = −0.145, *P* < 0.005); (ReHo in right visual association cortex–leptin: *r* = −0.270, *P* = 0.005; leptin–BMI: *r* = 0.710, *P* < 0.001 and ReHo in right visual association cortex–BMI: *r* = −0.193, *P* < 0.001); (ReHo in left visual association cortex–leptin: *r* = −0.306, *P* = 0.001; leptin–BMI: *r* = 0.710, *P* < 0.001 and ReHo in left visual association cortex–BMI: *r* = −0.181, *P* < 0.001); (ReHo in left dorsolateral prefrontal cortex–leptin: *r* = 0.233, *P* = 0.016; leptin–BMI: *r* = 0.710, *P* < 0.001; ReHo in left dorsolateral prefrontal cortex–BMI: *r* = 0.136, *P* = 0.008; Fig. 2; Tables S4–S7).

### Confounding effects

After adjusting for potential confounding factors (i.e. illness duration, the first episode and medication status), these results remained consistent with our main findings (see Supplementary Materials and Table S8).

### Mediation analyses

**Fig. 3:**
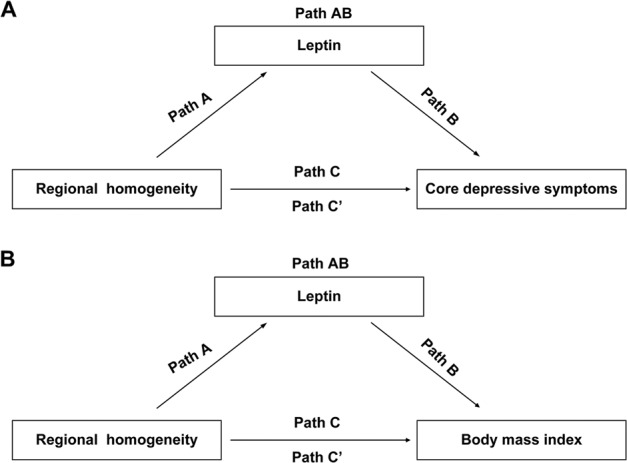
Mediation models. **a** Path A represents the association between ReHo values in the primary somatosensory and visual association cortices and leptin. Path B represents the association between leptin and core depressive symptoms. Path C represents the association between ReHo values and core depressive symptoms. Path C represents the total effect of ReHo values on core depressive symptoms; and Path C’ shows the association between ReHo values and core depressive symptoms not through leptin; whereas path AB represents the indirect effect of ReHo values on core depressive symptoms mediated by leptin. **b** Path A represents the association between ReHo values in the primary somatosensory and visual association cortices and leptin. Path B represents the association between leptin and BMI. Path C represents the association between ReHo values and BMI. Path C represents the total effect of ReHo values on BMI; and Path C’ shows the association between ReHo values and BMI not through leptin; whereas path AB represents the indirect effect of ReHo values on BMI mediated by leptin. BMI body mass index, ReHo regional homogeneity.

In mediation analyses, ReHo in right visual association and right primary somatosensory cortices had a significant negative effect on plasma leptin level (path A: 95% CI, −38,242.653 to −2912.998; 95% CI, −31,975.787 to −3903.892 respectively), whereas plasma leptin level had no significant effect on psychological and somatic depressive symptoms (path B: 95% CI, −0.0002 to 0.000; 95% CI, −0.0001 to 0.000). Total effect of the ReHo in right visual association cortex on psychological depressive symptoms was significant (path C: 95% CI, 2.081 to 18.043). Likewise, the total effect of the ReHo in right primary somatosensory cortex on somatic depressive symptoms was also significant (path C: 95% CI, 0.063 to 9.528). After adding the plasma leptin level as a mediator, the direct effect was no longer significant (path C’: 95% CI, −0.869 to 16.617; 95% CI, −0.821 to 8.681), whereas the indirect path with plasma leptin level as a mediator was significant (path AB: 95% CI, 0.419 to 5.590; 95% CI, 0.340 to 2.309, respectively; Fig. 3a, Tables S9–S10).

ReHo values in the right primary somatosensory, right visual association and left visual association cortices had a significant negative relationship with leptin (path A: 95% CI, −31,975.787 to −3903.892; 95% CI, −38,242.653 to −2912.998; 95% CI, −45,302.334 to −9777.606, respectively). Furthermore, leptin had a significant positive effect on BMI (path B, 95% CI, 0.0002 to 0.0003). The total effect of the ReHo in the right primary somatosensory, right visual association and left visual association cortices on BMI was significant (path C: 95% CI, −14.761 to −2.048; 95% CI, −18.299 to −4.879 and 95% CI, −21.544 to −6.692, respectively). After adding leptin as a mediator, the direct effect from the ReHo in the right primary somatosensory cortex on BMI was no longer significant (path C’: 95% CI, −10.092 to 0.092), but the direct effect from right and left visual association cortices on BMI was significant (95% CI, −11.687 to −1.882; 95% CI, −14.264 to −2.371, respectively), whereas the indirect path via leptin was significant (path AB: 95% CI, −8.546 to −1.305; 95% CI, −9.197 to −0.835 and 95% CI, −11.567 to −2.595, respectively). No other significant mediation effects were identified (Fig. 3b and Tables S10–S11).

## Discussion

In this study, we employed a novel approach to assess the neurobiological underpinning of depressive symptoms across MPDs in a large sample from a single site. We are not aware of other such studies. Here we observed the following: (1) lower ReHo in the primary sensory cortex (including primary somatosensory, auditory and visual cortices) and visual association cortex and higher ReHo in the prefrontal cortex (involving orbitofrontal and dorsolateral prefrontal cortices) and angular gyrus and the plasma leptin levels elevated in patients with MPDs. (2) ReHo values (right primary somatosensory cortex, bilateral visual association cortices and left dorsolateral prefrontal cortex), plasma leptin levels, HAMD scores (psychological and somatic depressive symptoms) and BMI in MPDs were significantly associated. The psychological and somatic depressive symptoms displayed similar results, and we further combined them together as core depressive symptoms. (3) Leptin mediated both the association between ReHo alterations (primary somatosensory and visual association cortices) and core depressive symptoms as well as BMI. These findings are consistent with an expanding literature implicating primary somatosensory cortex^5,38,39^, visual association cortex^40,41^ and leptin^25,42^ in depression and further suggesting that depressive symptoms are dimensional features across MPDs.

Utilising a transdiagnostic approach, the extent of overlap and distinct alterations in ReHo values among SCZ, BPD and MDD were examined. Notably, common alterations represented 86% of the total regional values that showed significant differences in the four-group analysis, suggesting the presence of common neuroanatomical substrates in SCZ, BPD and MDD. As distinct differences may have been less prominent among the three diagnostic categories, this unclear boundary further supports the need for research using transdiagnostic designs. Moreover, these differences were graded, with greatest alterations in SCZ, followed by BPD, and then MDD, which mirror the clinical severity and prognosis of three disorders. Our current findings are in line with previous GWAS^43^ and neuroimaging studies^17,44–46^. Taken together, this study provides critical insights into the biological basis of SCZ, BPD and MDD from a transdiagnostic perspective.

We found a lower ReHo within primary sensory and visual association cortices, while higher ReHo within the prefrontal cortex and angular gyrus in MPDs compared with the controls. The findings are consistent with our previous reports as well as the reports of others^17,18,45^, suggesting an imbalance between network segregation and integration across MPDs^17^. Consistent with a previous study^18^, we observed ReHo alterations in primary sensory, visual association and orbital frontal cortices associated with depressive symptoms. The abovementioned brain regions are involved in emotion processing^38–40,47^. Primary somatosensory and visual association cortices play important roles in encoding somatosensory sensations and emotional recognition and regulation^39^. Previous authors had demonstrated the interaction of emotion and somatic symptoms in the somatosensory cortex^48^. Core depressive symptoms, including depressed mood, anhedonia and somatic symptoms, could arise from these abnormalities in emotion processing^47^. These results conform to a previous research^5,18,39^, suggesting that ReHo alterations in the primary somatosensory and visual association cortices are common neuroanatomical substrates across MPDs.

Furthermore, we also found an important relationship among lower ReHo in the primary somatosensory and visual association cortex, elevated leptin levels and depressive symptoms in patients with MPDs. Findings in mediation models implicate a causal role for elevated leptin levels in the pathophysiology of depression. Lower regional FC in these brain regions may implicate possible reduction in neuronal excitability, especially to decrease the activity of GABAergic neurons^49^. Interestingly, reduction of GABAergic neurons could process the signals arriving from the periphery such as leptin^50^. Leptin has been consistently associated with emotional processing in the brain^22,25,51^. Its receptors are widely distributed in emotional processing regions, such as primary somatosensory and visual association cortices^52^. Leptin also influences neural function in the emotional control of food intake and partly in weight by reducing the GABA release^25^. Thus elevated circulating leptin levels that we observed in patients with MPDs may have functional consequences for neuronal excitability. Collectively, our data suggest that functional abnormalities of the primary somatosensory and visual association cortices may be associated with elevated leptin levels at the periphery that contribute to depressive symptoms.

An intriguing aspect of our findings is the mediating effect of leptin on the association between ReHo alterations in primary somatosensory and visual association cortices and core depressive symptoms. The GABAergic deficits may be the biological mechanism underlying the leptin-mediated effect in depression^53^. Indeed, leptin may directly act on presynaptic GABAergic neurons to induce its mediated effect^54^.The plasticity of GABAergic neurons is critical in the development of the primary sensory and visual association cortices^39,41^. GABAergic reduction in the primary somatosensory and visual association cortices in depression have been documented^25,41,53,55^. Primary somatosensory and visual association cortices in depressive symptoms are essential, and leptin is an important link between ReHo alterations and core depressive symptoms, which may possibly relate to GABAergic neurons. Overall, our main findings demonstrated common neurobiological mechanisms for the leptin-mediated core depressive symptoms, suggesting a potential therapeutic target for depressive symptoms across MPDs.

In patients with MPDs, BMI was associated with ReHo alterations, leptin, and depressive symptoms, contrary to that in healthy controls. Interestingly, leptin also mediated the association between ReHo alterations in primary somatosensory cortex, visual association cortex and BMI. Moreover, BMI was associated with core depressive symptoms, such as depressed mood, weight changes, appetite and genital symptoms^56–60^. The underlying mechanism of this association may be related with the changes in the hypothalamic–pituitary–adrenal axis, glucocorticoid receptors and GABAergic system^58,61^. The current findings could expand our understanding of BMI as an objective measure to assess the severity of depressive symptoms^62,63^.

This study has some limitations. First, the cross-sectional design limits our interpretation of causal relationships. Thus we could not determine how the dynamic relationship among ReHo values, leptin and depressive symptoms changes. Future longitudinal research is needed to define the causal relationship and neurobiological mechanisms of depressive symptoms. Second, our study is limited by possible confounding effects from illness duration, medication, lifestyle and dietary habits. Future studies will aim to explore potential variations in illness duration. Third, this range could be reflective of the transdiagnostic continuum for depressive symptomatology. Finally, an updated version of the volume-based ReHo has been developed on the cortical surface and demonstrated a more biologically plausible validity^12,14,15^. In our future work, we will validate and investigate the reproducibility of the present findings across methods, study designs and centres.

## Conclusion

Transdiagnostic associations existed between ReHo, leptin, depressive symptoms and BMI, and leptin is an important mediator among ReHo alterations in primary somatosensory and visual association cortices, core depressive symptoms and BMI. Our findings illustrate common neuroanatomical substrates and neurobiological mechanisms for the leptin-mediated core depressive symptoms and ultimately provide a potential therapeutic target for dimensional depressive symptoms across MPDs.

## Supplementary information

Supplementary tables

Supplementary Figure S1

Supplementary Figure S2
