# Supplementary material for: Applying dimensional psychopathology: transdiagnostic associations among regional homogeneity, leptin and depressive symptoms
Source: Transl Psychiatry. 2020 Jul 22;10:248. doi: 10.1038/s41398-020-00932-0 (PMC7376105; doi:10.1038/s41398-020-00932-0)
Supplement: Supplementary file 1 — Supplementary tables [file 41398_2020_932_MOESM1_ESM.doc]

**Applying dimensional psychopathology: transdiagnostic associations among** **regional homogeneity, leptin and depressive** **symptoms**

Yan-ge Wei, MD, PhD^1-3^, Jia Duan, MD, PhD^1, 3^, Fay Y. Womer, MD^4^, Yue Zhu, MD^1, 3^, Zhiyang Yin, MD, PhD^1, 3^ Lingling Cui, MD, PhD^1, 5^, Chao Li., PhD^1, 5^, Zhuang Liu, PhD^1, 6^, Shengnan Wei, MD, PhD^1, 5^, Xiaowei Jiang, MD, PhD^1, 5^, Yanbo Zhang, MD, PhD^7^, Xizhe Zhang, PhD^8, 9^, Yanqing Tang, MD, PhD^1, 3, 10 *^, Fei Wang, MD, PhD^1, 3, 5 *^

^1^ Brain Function Research Section, the First Affiliated Hospital of China Medical University,
Shenyang 110001, Liaoning, P.R. China

^2^ Henan Key Laboratory of Immunology and Targeted Drugs, School of Laboratory Medicine, Xinxiang Medical University, Xinxiang 453003, Henan, P.R. China

^3^ Department of Psychiatry, the First Affiliated Hospital of China Medical University, Shenyang 110001, Liaoning, P.R. China

^4^ Department of Psychiatry, Washington University School of Medicine, St. Louis, MO 63130, USA

^5^ Department of Radiology, the First Affiliated Hospital of China Medical University, Shenyang 110001, Liaoning, P.R. China

^6^ School of Public Health, China Medical University, Shenyang 110001, Liaoning, P.R. China

^7^ Department of Psychiatry, College of Medicine University of Saskatchewan Ellis Hall, Royal University Hospital, Saskatoon, SK S7N 0W8, Canada

^8^ Affiliated Nanjing Brain Hospital, Nanjing Medical University, Nanjing 210029, Jiangsu, P.R. China

^9^ School of Biomedical Engineering and Informatics, Nanjing Medical University, Nanjing 211166, Jiangsu, P.R. China

^10^ Department of Geriatrics, the First Affiliated Hospital of China Medical University, Shenyang 110001, Liaoning, P.R. China

^*^ These authors contributed equally to this work.

**Corresponding Author:**

Fei Wang, MD, PhD, Department of Psychiatry and Radiology, The First Affiliated Hospital, China Medical University, 155 Nanjing North Street, Shenyang 110001, Liaoning, PR China. Phone: +8624 8328 3405. Fax: +8624 8328 3405. Email: feiwangster@yeah.net.

Yanqing Tang, MD, PhD, Department of Psychiatry and Geriatrics, The First Affiliated Hospital, China Medical University,155 Nanjing North Street, Shenyang 110001, Liaoning, PR China. Phone: +8624 8328 3405. Fax: +8624 8328 3405. Email: [tangyanqing@cmu.edu.cn](mailto:tangyanqing@cmu.edu.cn)

Supplementary Content

**Supplementary Methods.**

**Supplementary Results.**

Figure S1. Group differences in regional homogeneity values in patients with schizophrenia, bipolar disorder, major depressive disorder and healthy controls.

Figure S2. Group differences in plasma leptin levels in patients with schizophrenia, bipolar disorder, major depressive disorder and healthy controls.

Table S1. Demographic and clinical characteristics in patients with major psychiatric disorders.

Table S2. Factor loadings of the HAMD.

Table S3. Correlations between regional homogeneity values, plasma leptin levels, and BMI in healthy controls.

Table S4. Correlations between regional homogeneity values and HAMD scores in patients with major psychiatric disorders.

Table S5. Correlations between HAMD scores and plasma leptin levels in patients with major psychiatric disorders.

Table S6. Correlations between regional homogeneity values and plasma leptin levels in patients with major psychiatric disorders.

Table S7. Correlations between regional homogeneity values, plasma leptin levels, HAMD scores, and BMI in patients with major psychiatric disorders.

Table S8. Effects of illness duration, first episode, medication status on regional homogeneity values and leptin levels in major psychiatric disorders.

Table S9. Mediation analysis of the relationship between regional homogeneity values and HAMD score, leptin as mediator.

Table S10. Mediation Results in patients with major psychiatric disorders.

Table S11. Mediation analysis of the relationship between regional homogeneity values and BMI, leptin as mediator.

**Supplementary** **Material**

**Supplementary Methods**

*Measurement of plasma leptin levels*

Blood samples (5 ml) were collected and centrifuged at 2,000 rpm for 10 minutes. The plasma was stored in aliquots at -80°C for leptin measurements. Samples were magnetically labeled using a human, magnetic, premixed, microparticle cocktail of antibodies (Kit Lot Number L120614). 50μL of the microparticle cocktail and 50μL of the standard or sample were added to each well of a microplate. Plates were then placed on a horizontal orbital microplate shaker set at 800 ± 50 rpm. After washing, 50μL of a diluted biotin-antibody cocktail was added to each well and the plates were incubated. After washing, 50μL diluted Streptavidin-PE was added to each well, and the plates were incubated. After washing, the microparticles were suspended by adding 100μL of wash buﬀer to each well. The plates were then incubated for 2 minutes on the shaker set at 800 ± 50rpm. The final sample was read within 90 minutes using a Luminex MAGPIX technology (SN, USA). Duplicate readings for each standard and sample were averaged, and the average blank Median Fluorescence Intensity (MFI) was subtracted. A standard curve was generated for each cytokine to convert the MFI into the corresponding leptin relative concentration. The sensitivity of the assay for leptin was 104.940 pg/ml (minimum detectable level). After obtaining the blood samples, all subjects underwent MRI within 24 hours.

*HAMD factor analyses*

We used AMOS 17.0 (SPSS Inc., Chicago, IL, USA) for CFA analyses. CFA by max likelihood method was used to ascertain the factor structure of HAMD. Several model fit indices were used to evaluate the goodness-of-fit of the model with the given dataset: the goodness of fit index (GFI), the normal fit index (NFI), Tucker-Lewis Index (TLI), the incremental fit index (IFI), the comparative fit index (CFI), the standardized root mean square residual (SRMR), and the root mean square error of approximation (RMSEA). The GFI, NFI, TLI, IFI, and CFI values above 0.90, and SRMR values below 0.08 are considered acceptable. Regarding the RMSEA, a value ≤ 0.10 indicates good model fit.

*Statistical analyses*

*Demographic and clinical characteristic analyses*

Analyses of demographic and clinical characteristics and plasma leptin levels were performed using analysis of covariance (ANCOVA) and chi-square tests in the SPSS statistics software. Statistical significance was determined by *P* < 0.05.

*ReHo alterations across diagnostic groups*

We have performed additional analyses to compare ReHo values showing significant differences between MPDs and healthy controls in SCZ vs. HC, BPD vs. HC, and MDD vs. HC. Statistical significance was defined as *P* < 0.05, Bonferroni corrected for multiple comparisons.

*Effects of illness duration on ReHo values and leptin levels*

Nonparametric Spearman’s correlation analyses (two-tailed) were performed to determine the relationship between illness duration and ReHo values that showed significant group differences as well as leptin in patients with MPDs. Statistical significance was set at *P* < 0.05, false discovery rate (FDR) corrected for multiple comparisons.

*Effects of the first episode and medication status on ReHo values and leptin levels*

We subdivided the patient group into two subgroups according to their clinical information: first-episode vs. relapsed subgroups and medicated vs. nonmedicated subgroups for the ReHo values and leptin levels. Then, two-sample t-tests were performed between each pair of subgroups. Statistical significance was determined using FDR-corrected *P* < 0.05.

*Mediation analyses across diagnostic groups*

Mediation analysis within each diagnostic group was performed using PROCESS for SPSS 22.0 with a 5000 bootstrap samples. Age, gender, mean FD and medication as covariates were served as the covariates in mediation analyses. Statistical significance was achieved when 95% confidence intervals (CI) did not include zero for the estimates of indirect effect.

**Supplementary Results**

*Demographic and clinical characteristic analyses*

The variation within each group of data was checked before the statistical analyses. No significant differences in age, gender, handedness, BMI and smoking among the SCZ, BPD, MDD, and healthy controls were observed. Detailed demographic and clinical characteristics of major depressive disorder (MDD), bipolar disorder (BPD), and schizophrenia (SCZ) groups are shown in Table S1.

*HAMD factor analyses*

The model fit indices were as follows: GFI = 0.934, NFI = 0.935, TLI = 0.944, IFI = 0.953, CFI = 0.953, AGFI = 0.911, SRMR = 0.033, and RMSEA = 0.060. CFA results indicated that this model had a good fit.

*Analyses of plasma leptin levels*

Comparing each patient group to healthy controls, post-hoc analysis showed that plasma leptin levels were higher in MDD, BPD, and SCZ groups. Specific leptin levels of the MDD, BPD, and SCZ groups are listed in Figure S2 and Table S1.

*ReHo value findings*

The SCZ, BPD, and MDD groups shared ReHo alterations in 86% of the total regions with significant four-group differences. Overlap regions included left primary somatosensory cortex, left primary auditory cortex, right primary visual cortex, bilateral orbital frontal cortices, bilateral dorsolateral prefrontal cortices, and right angular gyrus. Additionally, the SCZ and BPD groups, but not the MDD group, had three clusters of significant ReHo alternations when compared with HC. These clusters consisted of right primary somatosensory cortex, left visual association cortex, and left angular gyrus. Significant ReHo reduction in the right visual association cortex was observed in the SCZ group only when compared with HC. No significant differences in ReHo values were observed between the MDD and controls in any of the four clusters (Figure S1).

*Effects of illness duration on ReHo values and leptin levels*

Illness duration was significantly negatively correlated with ReHo values in the right primary somatosensory cortex ( r = -0.017, *P* = 0.031) and left visual association cortex( r = -0.168, *P* = 0.002), and was significantly positively correlated with ReHo values in the left angular gyrus ( r = 0.136, *P* = 0.012) in patients with MPDs (Table S8).

*Effects of the first episode and medication status on ReHo values and leptin levels*

No significant differences were observed for ReHo values and leptin levels, either between the first-episode and relapsed subgroups or between the medicated and nonmedicated subgroups (Table S8).

*Mediation analyses across diagnostic groups*

No significant mediation effects were identified for SCZ, BPD and MDD groups.

**Supplementary Figure Legends**

Figure S1. Cluster-based post hoc analyses in regional homogeneity values in patients with schizophrenia, bipolar disorder, major depressive disorder and healthy controls.

* Statistical significance was determined by Bonferroni-corrected *P* < 0.05.

BPD = bipolar disorder; HC = healthy controls; MDD= major depressive disorder; SCZ = schizophrenia

Figure S2. Group differences in plasma leptin levels in patients with schizophrenia, bipolar disorder, major depressive disorder and healthy controls.

*, Statistical significance was determined by Bonferroni-corrected *P* < 0.05.

BPD = bipolar disorder; HC = healthy controls; MDD= major depressive disorder; SCZ = schizophrenia.

**Supplementary Tables**

**Table S1. Demographic and clinical characteristics in patients with major psychiatric disorders.**

| Characteristic | Group; mean ± SD or no. (%) | | | |  |  |
| --- | --- | --- | --- | --- | --- | --- |
|  | Control  (n = 325) | MDD  (n = 153) | BPD  (n = 123) | SCZ  (n = 127) | Statistics | *P* value |
| Demographic characteristic | | | | | |  |
| Age, years | 29.24 (9.94) | 29.87 (8.90) | 28.11(8.56) | 27.13 (8.99) | 2.488 | 0.059 |
| Gender (male/female) | 132/193 | 44/109 | 51/72 | 48/79 | 7.120 | 0.068 |
| Right handedness | 303 (93%) | 135 (88%) | 119 (97%) | 106 (83%) | 11.174 | 0.083 |
| Weight^1^ | 62.41 (14.39) | 59.73 (11.60) | 65.09 (13.35) | 64.48 (13.35) | 4.315 | 0.005 |
| BMI (kg/m^2^) ^1^ | 23.76 (26.60) | 21.77 (3.65) | 22.89 (3.91) | 23.23 (4.04) | 0.404 | 0.751 |
| Smoking | 37/188 (20%) | 19/75 (25%) | 19/67 (28%) | 11/64 (17%) | 3.530 | 0.317 |
| Clinical characteristic | | | | | | |
| First episode, yes | - | 125 (82%) | 58 (47%) | 79 (62%) | - | - |
| Medication, yes | - | 83 (54%) | 86 (70%) | 90 (71%) | - | - |
| Antidepressant | - | 71 (17%) | 41 (10%) | 13 (3%) | - | - |
| Mood stabilizer | - | 1 (0.2%) | 63 (16%) | 6 (1%) | - | - |
| Antipsychotic | - | 9 (2%) | 46 (11%) | 69 (17%) | - | - |
| Duration, months | - | 22.12 (33.68) | 48.46 (65.73) | 30.44 (42.00) | - | - |
| HAMD^2^ | 1.27 (2.16) | 20.07 (9.84) | 11.54 (10.14) | 7.15 (6.10) | 260.998 | < 0.001 |
| YMRS^3^ | 0.25 (0.93) | 1.20 (3.15) | 5.89 (8.97) | 1.71 (4.23) | 46.076 | < 0.001 |
| BPRS^4^ | 18.42 (1.94) | 26.70 (7.21) | 25.57 (8.46) | 32.85 (11.67) | 112.986 | < 0.001 |
| Leptin (pg/ml) ^5^ | 5463.13 (4055.93) | 6033.64 (5243.52) | 9570.49 (8877.02) | 14510.21 (12885.72) | 12.299 | < 0.001 |
| Leptin (log) ^5^ | 3.57 (0.44) | 3.61 (0.43) | 3.81 (0.44) | 3.97 (0.46) | 7.19 | < 0.001 |

BMI = Body Mass Index; BPD = bipolar disorder; BPRS = Brief Psychiatric Rating Scale; HAMD = Hamilton Depression Scale; MDD = major depressive disorder; SCZ = schizophrenia; YMRS = Young Mania Rating Scale.

^1^ Control, n = 314; MDD, n = 143; BPD, n = 120; SCZ, n = 122.

^2^ Control, n = 303; MDD, n = 150; BPD, n = 120; SCZ, n = 104.

^3^Control, n = 298; MDD, n = 137; BPD, n = 118; SCZ, n = 82.

^4^Control, n = 237; MDD, n = 101; BPD, n = 79; SCZ, n =123.

^5^ Control, n = 83; MDD, n = 41; BPD, n = 42; SCZ, n = 28.

**Table S2. Factor loadings of the HAMD.**

| Items | Factor Loadings | | | |
| --- | --- | --- | --- | --- |
|  | Factor 1 | Factor 2 | Factor 3 | Factor 4 |
| Guilt | **.773** | .216 | .116 | .130 |
| Suicide | **.716** | .211 | .267 | .040 |
| Depressed mood | **.696** | .366 | .333 | .240 |
| Work and interests | **.658** | .359 | .290 | .241 |
| Hypochondria | **.587** | .040 | .314 | -.039 |
| Psychic anxiety | **.551** | .306 | .311 | .317 |
| Retardation | **.499** | .459 | .106 | .386 |
| Gastrointestinal symptoms | .275 | **.711** | .195 | .165 |
| Weight loss | .300 | **.679** | .102 | .080 |
| Genital symptoms | .012 | **.678** | .281 | .038 |
| General somatic symptoms | .427 | **.530** | .331 | .186 |
| Somatic anxiety | .378 | **.504** | .380 | .167 |
| Middle insomnia | .328 | .260 | **.763** | .193 |
| Late insomnia | .257 | .275 | **.755** | .079 |
| Early insomnia | .297 | .244 | **.753** | .179 |
| Insight | -.023 | .064 | .209 | **.847** |
| Agitation | .371 | .199 | .044 | **.602** |

HAMD = Hamilton Depression Scale.

Loadings in bold indicate the maximum loading of each item onto one of the four factors.

Factor 1 = psychological depressive symptoms.

Factor 2 = somatic depressive symptoms.

Factor 3 = insomnia

Factor 4 = mixed symptoms.

**Table S3. Correlations between regional homogeneity values, plasma leptin levels, and BMI in healthy controls.**

| Brain region | BMI | |
| --- | --- | --- |
|  | *r* | *P* |
| Right primary somatosensory cortex | 0.047 | 0.414 |
| Left primary somatosensory cortex | 0.025 | 0.661 |
| Left primary auditory cortex | 0.017 | 0.766 |
| Right primary visual cortex | 0.060 | 0.290 |
| Right visual association cortex | 0.035 | 0.543 |
| Left visual association cortex | -0.036 | 0.521 |
| Right orbital frontal cortex | -0.001 | 0.992 |
| Left orbital frontal cortex | 0.013 | 0.816 |
| Right dorsolateral prefrontal cortex | -0.055 | 0.338 |
| Left dorsolateral prefrontal cortex | -0.032 | 0.571 |
| Right angular gyrus | -0.046 | 0.414 |
| Left angular gyrus | -0.070 | 0.221 |
| Leptin | -0.009 | 0.926 |

BMI = Body Mass Index.

Correlation coefficients were statistically significant at *P* < 0.05 false discovery rate (FDR) corrected for multiple comparisons. Age, gender, and mean framewise displacement were treated as covariates.

**Table S4. Correlations between regional homogeneity values and HAMD scores in patients with major psychiatric disorders.**

| Brain region | HAMD total score | | Psychological depressive symptoms | | Somatic depressive symptoms | | Insomnia | | Mixed symptoms | |
| --- | --- | --- | --- | --- | --- | --- | --- | --- | --- | --- |
|  | *r* | *P* | *r* | *P* | *r* | *P* | *r* | *P* | *r* | *P* |
| Right primary somatosensory cortex | 0.096 | 0.065 | 0.129 | 0.010* | 0.114 | 0.023* | 0.134 | 0.007* | -0.004 | 0.929 |
| Left primary somatosensory cortex | 0.084 | 0.109 | 0.100 | 0.046 | 0.135 | 0.007* | 0.133 | 0.008* | 0.021 | 0.678 |
| Left primary auditory cortex | 0.120 | 0.021* | 0.103 | 0.040 | 0.124 | 0.013* | 0.095 | 0.058 | 0.040 | 0.430 |
| Right primary visual cortex | 0.016 | 0.766 | 0.043 | 0.397 | 0.054 | 0.283 | 0.091 | 0.069 | 0.030 | 0.546 |
| Right visual association cortex | 0.072 | 0.167 | 0.115 | 0.022* | 0.094 | 0.059 | 0.115 | 0.021* | 0.017 | 0.738 |
| Left visual association cortex | 0.060 | 0.250 | 0.084 | 0.094 | 0.094 | 0.060 | 0.127 | 0.011* | 0.012 | 0.804 |
| Right orbital frontal cortex | -0.048 | 0.357 | -0.073 | 0.146 | -0.115 | 0.022* | -0.071 | 0.158 | 0.014 | 0.786 |
| Left orbital frontal cortex | -0.038 | 0.471 | -0.065 | 0.198 | -0.082 | 0.100 | -0.080 | 0.111 | 0.046 | 0.358 |
| Right dorsolateral prefrontal cortex | -0.020 | 0.700 | 0.012 | 0.808 | -0.044 | 0.379 | -0.071 | 0.159 | -0.043 | 0.388 |
| Left dorsolateral prefrontal cortex | 0.008 | 0.883 | -0.013 | 0.799 | -0.048 | 0.344 | -0.080 | 0.110 | -0.005 | 0.917 |
| Right angular gyrus | 0.013 | 0.803 | 0.012 | 0.812 | -0.024 | 0.634 | -0.025 | 0.618 | 0.012 | 0.814 |
| Left angular gyrus | -0.040 | 0.440 | -0.038 | 0.454 | -0.101 | 0.045 | -0.091 | 0.069 | 0.028 | 0.577 |

HAMD = Hamilton Depression Scale.

*Correlation coefficients were statistically significant at *P* < 0.05 false discovery rate (FDR) corrected for multiple comparisons. Age, gender, mean framewise displacement (FD), and medication were treated as covariates.

**Table S5. Correlations between HAMD scores and** **plasma leptin levels in patients with major psychiatric disorders.**

| Clinical variables | Leptin | |
| --- | --- | --- |
|  | *r* | *P* |
| HAMD total score | -0.305 | 0.002* |
| Psychological depressive symptoms | -0.261 | 0.007* |
| Somatic depressive symptoms | -0.317 | 0.001* |
| Insomnia | -0.190 | 0.050 |
| Mixed symptoms | 0.010 | 0.916 |

HAMD = Hamilton Depression Scale.

*Correlation coefficients were statistically significant at *P* < 0.05 false discovery rate (FDR) corrected for multiple comparisons. Age, gender, mean framewise displacement, and medication were treated as covariates.

**Table S6. Correlations between regional homogeneity values and** **plasma leptin levels** **in patients with major psychiatric disorders.**

| Brain region | Leptin | |
| --- | --- | --- |
|  | *r* | *P* |
| Right primary somatosensory cortex | -0.245 | 0.011* |
| Left primary somatosensory cortex | -0.180 | 0.063 |
| Left primary auditory cortex | -0.192 | 0.047 |
| Right primary visual cortex | 0.200 | 0.039 |
| Right visual association cortex | -0.270 | 0.005* |
| Left visual association cortex | -0.306 | 0.001* |
| Right orbital frontal cortex | 0.101 | 0.299 |
| Left orbital frontal cortex | 0.176 | 0.070 |
| Right dorsolateral prefrontal cortex | 0.077 | 0.431 |
| Left dorsolateral prefrontal cortex | 0.233 | 0.016* |
| Right angular gyrus | 0.063 | 0.517 |
| Left angular gyrus | -0.032 | 0.745 |

*Correlation coefficients were statistically significant at *P* < 0.05 false discovery rate (FDR) corrected for multiple comparisons. Age, gender, mean framewise displacement, and medication were treated as covariates.

**Table S7. Correlations between regional homogeneity values, plasma leptin levels, HAMD scores, and BMI in patients with major psychiatric disorders.**

| Brain region | BMI | |
| --- | --- | --- |
|  | *r* | *P* |
| Right primary somatosensory cortex | -0.145 | 0.005* |
| Left primary somatosensory cortex | -0.137 | 0.007* |
| Left primary auditory cortex | -0.207 | 0.000* |
| Right primary visual cortex | -0.139 | 0.007* |
| Right visual association cortex | -0.193 | 0.000* |
| Left visual association cortex | -0.181 | 0.000* |
| Right orbital frontal cortex | 0.150 | 0.003* |
| Left orbital frontal cortex | 0.124 | 0.016* |
| Right dorsolateral prefrontal cortex | 0.153 | 0.003* |
| Left dorsolateral prefrontal cortex | 0.136 | 0.008* |
| Right angular gyrus | 0.020 | 0.699 |
| Left angular gyrus | 0.049 | 0.339 |
| Leptin | 0.710 | < 0.001* |
| HAMD total score | -0.210 | < 0.001* |
| Psychological depressive symptoms | -0.184 | < 0.001* |
| Somatic depressive symptoms | -0.194 | < 0.001* |
| Insomnia | -0.188 | < 0.001* |
| Mixed symptoms | -0.122 | 0.017* |

BMI = Body Mass Index.

*Correlation coefficients were statistically significant at *P* < 0.05 false discovery rate (FDR) corrected for multiple comparisons. Age, gender, mean framewise displacement, and medication were treated as covariates.

**Table S8. Effects of illness duration, first episode, medication status on regional homogeneity values and leptin levels in major psychiatric disorders.**

| Variables | Duration  (months) | | first episode  (yes vs. no) | | medication  (on / off) | |
| --- | --- | --- | --- | --- | --- | --- |
|  | r | *P* | T | *P* | T | *P* |
| Right primary somatosensory cortex | -0.117 | 0.031* | -2.317 | 0.021 | 1.288 | 0.198 |
| Left primary somatosensory cortex | -0.064 | 0.239 | -0.805 | 0.421 | .755 | 0.451 |
| Left primary auditory cortex | -0.104 | 0.054 | -1.987 | 0.048 | 1.735 | 0.084 |
| Right primary visual cortex | -0.008 | 0.876 | 0.293 | 0.770 | 1.587 | 0.113 |
| Right visual association cortex | -0.059 | 0.281 | -0.339 | 0.735 | 1.712 | 0.088 |
| Left visual association cortex | -0.168 | 0.002* | -2.688 | 0.007 | 0.940 | 0.348 |
| Right orbital frontal cortex | 0.108 | 0.045 | 2.858 | 0.004 | 0.114 | 0.909 |
| Left orbital frontal cortex | 0.035 | 0.516 | 1.802 | 0.072 | 1.612 | 0.108 |
| Right dorsolateral prefrontal cortex | 0.096 | 0.077 | 0.485 | 0.628 | -1.574 | 0.116 |
| Left dorsolateral prefrontal cortex | -0.005 | 0.924 | -0.378 | 0.705 | -1.934 | 0.054 |
| Right angular gyrus | 0.046 | 0.396 | 0.464 | 0.643 | -1.645 | 0.101 |
| Left angular gyrus | 0.136 | 0.012* | 1.072 | 0.284 | -1.098 | 0.273 |
| Leptin | 0.033 | 0.767 | 0.721 | 0.472 | 0.111 | 0.912 |

*Statistical significance was set at *P* < 0.05 (FDR correction).

**Table S9. Mediation analysis of the relationship between regional homogeneity values and HAMD score, leptin as mediator.**

| Model | Effect type (mediator variable: leptin) | Point estimate* | SE | 95% CI |
| --- | --- | --- | --- | --- |
| A | Outcome variable: psychological depressive symptoms |  |  |  |
|  | Mediation effect (ReHo in right primary somatosensory cortex-leptin-psychological depressive symptoms) | 2.032 | 1.018 | 0.535, 4.697 |
|  | Direct effect (ReHo in right primary somatosensory cortex-psychological depressive symptoms) | 7.553 | 4.794 | -1.978, 17.055 |
|  | Total effect | 9.156 | 4.803 | -0.363, 18.675 |
| B | Outcome variable: psychological depressive symptoms |  |  |  |
|  | Mediation effect (ReHo in right visual association cortex-leptin-psychological depressive symptoms) | 2.152 | 1.170 | 0.419, 5.590 |
|  | Direct effect (ReHo in right visual association cortex-psychological depressive symptoms) | 7.874 | 4.411 | -0.869, 16.617 |
|  | Total effect | 10.062 | 4.027 | 2.081, 18.043 |
| C | Outcome variable: somatic depressive symptoms |  |  |  |
|  | Mediation effect (ReHo in right primary somatosensory cortex-leptin-somatic depressive symptoms) | 1.098 | 0.487 | 0.340, 2.309 |
|  | Direct effect (ReHo in right primary somatosensory cortex -depressive symptoms) | 3.930 | 2.397 | -0.821, 8.681 |
|  | Total effect | 4.796 | 2.388 | 0.633, 9.528 |

ReHo = regional homogeneity. 95% CI, confidence interval of the point estimate.

*Point estimate, estimate of the size of the effect. Statistical significance was achieved when 95% confidence intervals (CI) did not include zero for the estimates of indirect effect. Models were adjusted for age, gender, mean framewise displacement, and medication.

**Table S10. Mediation Results in patients with major psychiatric disorders.**

| X-M-Y | Path A | Path B | Path C | Path C’ | Path AB |
| --- | --- | --- | --- | --- | --- |
| Right visual association cortex-leptin- psychological depressive symptoms | −38242.653,  −2912.998 | −0.0002,  0.000 | 2.081,  18.043 | −0.869,  16.617 | 0.419,  5.590 |
| Right primary somatosensory cortex-leptin- somatic depressive symptoms | −31975.787,  −3903.892 | −0.0001,  0.000 | 0.063,  9.528 | −0.821,  8.681 | 0.340,  2.309 |
| Right primary somatosensory cortex- leptin-BMI | −31975.787,  −3903.892 | 0.0002,  0.0003 | −14.761, −2.048 | −10.092,  0.092 | −8.546, −1.305 |
| Right visual association corte-leptin-BMI | −38242.653,  −2912.998 | 0.0002,  0.0003 | −18.299, −4.879 | −11.687, −1.882 | −9.197, −0.835 |
| left visual association cortex-leptin-BMI | −45302.334,  −9777.606 | 0.0002,  0.0003 | −21.544, −6.692 | −14.264, −2.371 | −11.567, −2.595 |

X-M-Y denotes the relationship among the independent variable (X), mediator (M), and dependent variable (Y).

Statistical significance was achieved when 95% confidence intervals (CI) did not include zero for the estimates of indirect effect. Models were adjusted for age, gender, mean framewise displacement, and medication.

**Table S11. Mediation analysis of the relationship between regional homogeneity values and BMI, leptin as mediator.**

| Model | Effect type (mediator variable: leptin) | Point estimate* | SE | 95% CI |
| --- | --- | --- | --- | --- |
| A | Outcome variable: BMI |  |  |  |
|  | Mediation effect (ReHo in right primary somatosensory cortex-leptin-BMI) | -4.318 | 1.796 | -8.546, -1.305 |
|  | Direct effect (ReHo in right primary somatosensory cortex-BMI) | -5.000 | 2.569 | -10.092, 0.092 |
|  | Total effect | -8.404 | 3.207 | -14.761, -2.048 |
| B | Outcome variable: BMI |  |  |  |
|  | Mediation effect (ReHo in right visual association cortex-leptin-BMI) | -4.726 | 2.105 | -9.197, -0.835 |
|  | Direct effect (ReHo in right visual association cortex-BMI) | -6.784 | 2.473 | -11.687, -1.882 |
|  | Total effect | -11.589 | 3.386 | -18.299, -4.879 |
| C | Outcome variable: BMI |  |  |  |
|  | Mediation effect (ReHo in left visual association cortex -leptin-BMI) | -6.318 | 2.203 | -11.567, -2.945 |
|  | Direct effect (ReHo in left visual association cortex-BMI) | -8.318 | 3.000 | -14.264, -2.371 |
|  | Total effect | -14.118 | 3.747 | -21.544, -6.692 |
| D | Outcome variable: BMI |  |  |  |
|  | Mediation effect (ReHo in left dorsolateral prefrontal cortex-leptin-BMI) | 4.222 | 3.823 | -2.460, 12.596 |
|  | Direct effect (ReHo in left dorsolateral prefrontal cortex-BMI) | 8.212 | 4.212 | -0.140, 16.565 |
|  | Total effect | 16.566 | 6.476 | 3.756, 29.406 |

ReHo = regional homogeneity. 95% CI, confidence interval of the point estimate.

*Point estimate, estimate of the size of the effect. Statistical significance was achieved when 95% confidence intervals (CI) did not include zero for the estimates of indirect effect. Models were adjusted for age, gender, mean framewise displacement, and medication.
