# Supplementary figures and images for: Applying dimensional psychopathology: transdiagnostic associations among regional homogeneity, leptin and depressive symptoms
Source: Transl Psychiatry. 2020 Jul 22;10:248. doi: 10.1038/s41398-020-00932-0 (PMC7376105; doi:10.1038/s41398-020-00932-0)

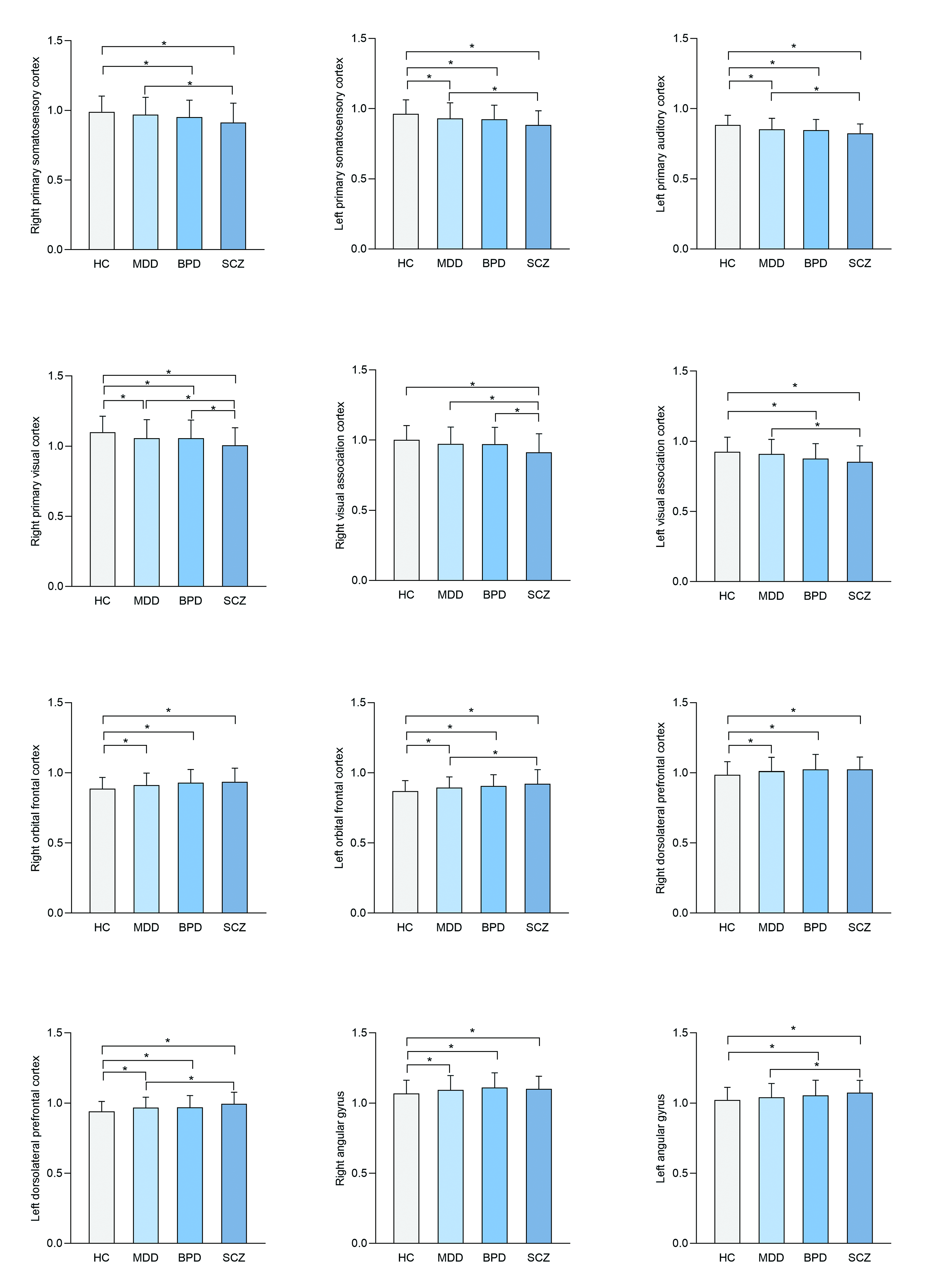

Supplement: Supplementary file 2 — Supplementary Figure S1 [file 41398_2020_932_MOESM2_ESM.tif]

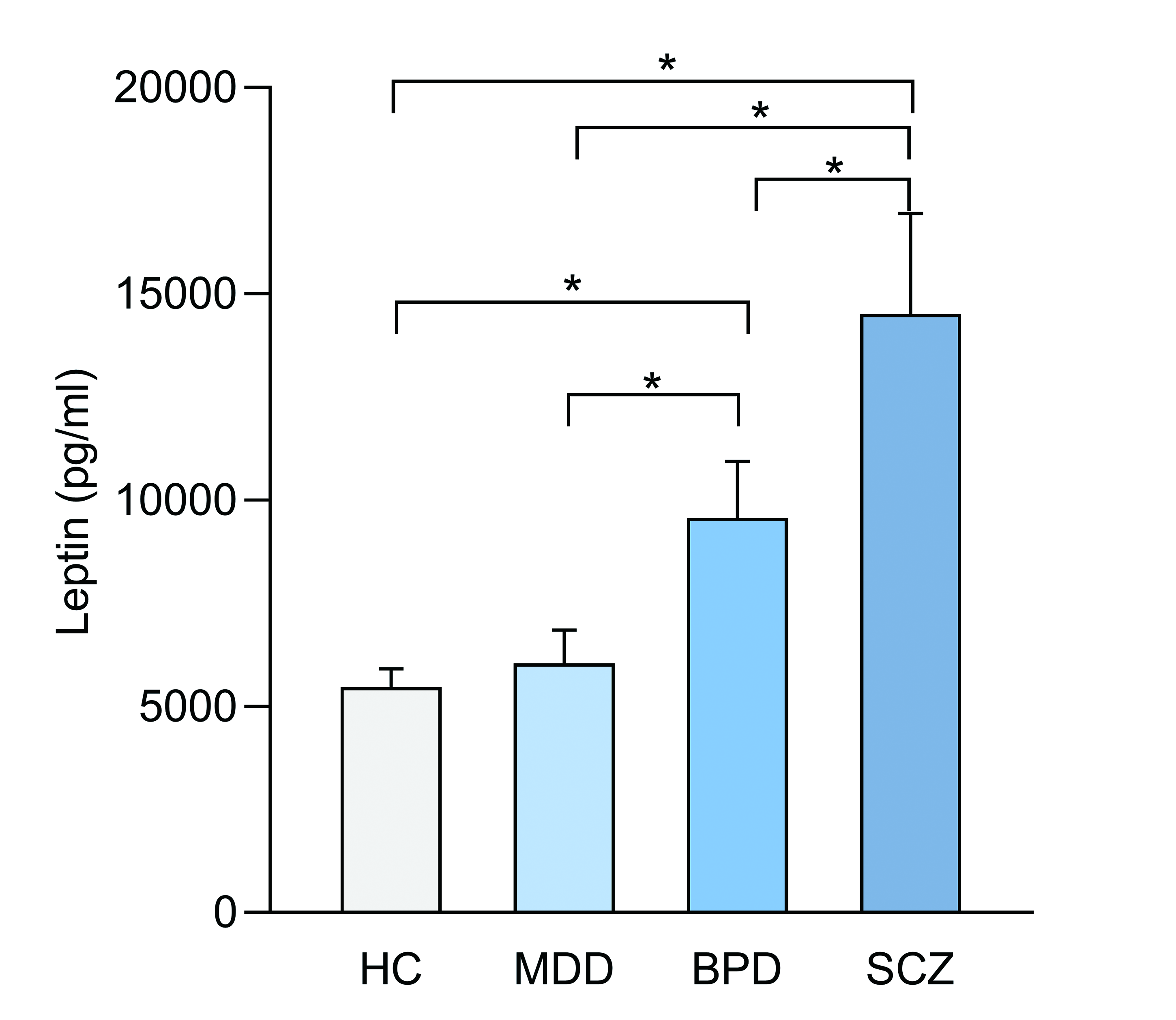

Supplement: Supplementary file 3 — Supplementary Figure S2 [file 41398_2020_932_MOESM3_ESM.tif]
